# Detection of differentially methylated regions from whole-genome bisulfite sequencing data without replicates

**DOI:** 10.1093/nar/gkv715

**Published:** 2015-07-15

**Authors:** Hao Wu, Tianlei Xu, Hao Feng, Li Chen, Ben Li, Bing Yao, Zhaohui Qin, Peng Jin, Karen N. Conneely

**Affiliations:** 1 Department of Biostatistics and Bioinformatics, Rollins School of Public Health, Emory University. 1518 Clifton Road. Atlanta, GA 30322, USA; 2 Department of Mathematics and Computer Science, Emory University. 400 Dowman Dr., W401. Atlanta, GA 30322, USA; 3 Department of Human Genetics, School of Medicine, Emory University. 615 Michael Street, Atlanta, GA 30322, USA

## Abstract

DNA methylation is an important epigenetic modification involved in many biological processes and diseases. Recent developments in whole genome bisulfite sequencing (WGBS) technology have enabled genome-wide measurements of DNA methylation at single base pair resolution. Many experiments have been conducted to compare DNA methylation profiles under different biological contexts, with the goal of identifying differentially methylated regions (DMRs). Due to the high cost of WGBS experiments, many studies are still conducted without biological replicates. Methods and tools available for analyzing such data are very limited.

We develop a statistical method, DSS-single, for detecting DMRs from WGBS data without replicates. We characterize the count data using a rigorous model that accounts for the spatial correlation of methylation levels, sequence depth and biological variation. We demonstrate that using information from neighboring CG sites, biological variation can be estimated accurately even without replicates. DMR detection is then carried out via a Wald test procedure. Simulations demonstrate that DSS-single has greater sensitivity and accuracy than existing methods, and an analysis of H1 versus IMR90 cell lines suggests that it also yields the most biologically meaningful results. DSS-single is implemented in the Bioconductor package DSS.

## INTRODUCTION

DNA methylation is a covalent epigenetic modification on the 5-carbon position of cytosine that plays important roles in regulating gene expression. Methylation of cytosine at promoter regions represses gene expression by interfering with the binding of transcription factors (TFs) or binding proteins that promote transcription (1). DNA methylation participates in many basic biological processes such as development and aging, and can be dysregulated in human diseases such as cancer (2–5). Because of its regulatory potential, studies of DNA methylation are of great interest. Results from these studies enhance the understanding of epigenetic mechanisms of many basic biological processes and disease etiologies.

Recent developments in high-throughput technologies such as second-generation sequencing have revolutionized the field by enabling genome-wide profiling of DNA methylation. Bisulfite conversion of DNA followed by high-throughput sequencing (also known as ‘Bisulfite sequencing’ or ‘BS-seq’) (6) allows measurement of DNA methylation at single CpG resolution, and has quickly become the technology of choice in DNA methylation studies. Data generated from whole-genome BS-seq (WGBS) experiments enable the comparison of genome-wide DNA methylation profiles under different biological contexts. Genomic regions showing different levels of DNA methylation under distinct biological conditions are termed ‘differentially methylated regions’ (DMRs). DMRs can provide insights into many important biological processes and human diseases. In addition, identification of DMRs between patients and unaffected individuals could lead to development of putative epigenetic biomarkers for early detection and diagnosis. For these reasons, there is great demand for methods for optimal detection of DMRs from WGBS data, where the goal is to define genomic regions that show statistically significant differences in methylation levels between biological conditions. A number of statistical methods and computational tools have been developed recently, including BSmooth (7), MethylKit (8), GBSA (9), BiSeq (10), DSS (11), MOABS (12), DMAP (13), MethylSig (14) and Bisulfighter (15).

Similar to other sequencing experiments, the raw data from WGBS experiments are short sequence reads. After alignment and data processing, the data can be summarized as a pair of counts for each CpG site: the number of reads showing methylation at this site and the total number of reads covering the site. The ratio of these two numbers provides an estimate of the methylation level at a CpG site. WGBS data possess several unique characteristics that should guide the design of any rigorous method to detect DMRs. We describe each of these characteristics below.

First, genome-wide methylation levels are characterized by strong spatial correlation (1,6). Proper utilization of the information from neighboring CpG sites can help improve estimation of methylation levels at each CpG site, and hence improve DMR detection. To incorporate this information, it is often assumed that the underlying true methylation levels can be represented by a smooth curve genome-wide, and smoothing techniques are used to estimate the curve (7). An additional advantage of smoothing is that it can help detect relatively longer DMRs that could be fragmented into smaller pieces if the spatial correlation is ignored.

Second, the read depth of the CpG sites provides information on precision that can be exploited to improve statistical tests for DMR detection. Methods that directly test the estimated methylation proportions (such as the *t*-test procedure implemented in BSmooth) lose information by ignoring the uncertainty in the point estimation. Moreover, some methods perform arbitrary filtering of sites with low depth (8), which results in loss of data. To make full use of the available information, read depth must be taken into account. Among the existing methods, the Wald test (11), likelihood ratio test (14) and CDIF (12) incorporate the depth information in DMR detection.

Finally, the variance among biological replicates provides information necessary for a valid statistical test to detect DMRs. Ignoring this variance—for example, aggregating the counts from replicates and applying Fisher's exact test (8,13)—can lead to false positives. BSmooth (7) avoids aggregating across replicates by performing a two-group *t*-test across conditions using the estimated methylation proportions, and thus directly computes the within-group variances. More recent approaches model the BS-seq count data using a beta-binomial distribution (11,14), which captures the biological variance through the dispersion parameters of the beta distribution. Estimation of the dispersion parameters is not a trivial task for the same reason plaguing differential expression analyses of RNA-seq data: the limited number of replicates possible within most budgets will often lead to unstable estimates. Borrowing ideas from the methods developed for RNA-seq (16,17), an empirical Bayes estimation procedure has been proposed and demonstrated to have good performance (11). An important requirement for these methods is that biological replicates are needed in order to estimate within-group variance. However, since WGBS experiments are still very expensive, many experiments performed to date only have a single replicate per condition (18–21). In this case, the typical solution is to ignore biological variance and perform naïve analyses based on the differences in estimated methylation levels or Fisher's exact test. These approaches implicitly assume that biological variance is constant across the genome, which is not true since it has been shown that there is substantial variation in the dispersion of methylation data genome-wide (3). Although this problem is often considered insurmountable, we will show below that even without replicates, the biological variance can still be estimated.

We argue that a good DMR detection method must account for the three characteristics described above: spatial correlation, read depth and biological variation. Of currently available methods, none account for all three characteristics in a statistically rigorous way when there is no biological replicate. In this work we propose a flexible, efficient and comprehensive method for DMR detection in two-group comparisons for WGBS data without replicates. We extend our previously proposed method (11) by incorporating spatial correlation into the model. Under our model specification, within group variance can be estimated even without replicates. This is achieved by combining data from nearby CpG sites and using them as ‘pseudo-replicates’ to estimate biological variance at specific locations. We perform extensive simulation and real data analyses, and demonstrate that our proposed method provides greater sensitivity, accuracy and biological plausibility compared with existing methods. This method, DSS-single (Dispersion Shrinkage for Sequencing data with single replicates) is now implemented in the DSS Bioconductor package.

## MATERIALS AND METHODS

### Modeling whole genome BS-seq data

For WGBS data from two groups and one replicate in each group, we use the following notation: At the *i*th CpG site and *j*th treatment group (*j = 1, 2*), let *X_ij_* be the count of methylated reads, and let *N_ij_* be the total read count. Denote the underlying ‘true’ methylation proportion by *p_ij_*. We have previously shown that it is reasonable to model *X_ik_* as a beta-binomial distribution, which captures both the biological and technical variation in the counts (11). The beta distribution is parameterized by mean (*μ_ij_*) and dispersion (*φ_ij_*), where *φ_ij_* represents the biological variance among replicates in the same treatment group. Further, a log-normal prior is imposed on *φ_ij_* in order to borrow information from all CpG sites in estimating the site-specific dispersions. To incorporate the spatial correlation in methylation levels, we assume that the underlying mean of the beta distribution, *μ_ij_*, varies smoothly across the genome. That is, we assume $\mu _{ij} = f_j (l_i )$, where *l_i_* denotes the genomic coordinate of the *i*th CpG site, and *f_j_* is a smooth function. Putting all of these pieces together, the data generated from WGBS experiments can be described by the following hierarchical model:

\begin{equation*} X_{ij} |N_{ij} ,p_{ij} \sim Binomial(N_{ij} ,p_{ij} ) \end{equation*}

\begin{equation*} p_{ij} |\mu _{ij} ,\phi _{ij} \sim Beta(\mu _{ij} ,\phi _{ij} ) \end{equation*}

\begin{equation*} \phi _{ij} \sim \log - normal(m_{j0} ,r_{j0}^2 ) \end{equation*}

\begin{equation*} \mu _{ij} = f_j (l_i ) \end{equation*}

This is a comprehensive model that captures all three of the important characteristics in the WGBS-seq data discussed above. The binomial distribution captures the random sampling process of the BS-seq experiment, the beta distribution models the biological variation among replicates, and the smooth function accounts for the spatial correlation among nearby CpG sites. The log-normal prior of the dispersion combines information from CpG sites genome-wide, which provides a basis for information sharing and helps the estimation of dispersion.

### Smoothing procedure

We use a simple moving average procedure of the collapsed counts to estimate *f_j_*. Specifically, at the *i*th CpG site, we estimate the mean by $\hat \mu _{ij} = {\sum\nolimits_{l \in S_i } {X_{lj}}}\left/{\sum\nolimits_{l \in S_i } {N_{lj} }}\right.$, where *S* is a set of CpG sites within a user-defined window of size *w* (500 base pairs by default), e.g. $S_i = \{ m:|l_m - l_i | < w\}$. We will show below that the simple procedure performs almost as well as more complicated, spline-based smoothing from BSmooth, yet it is much more computationally efficient.

### Dispersion estimation

With $\hat \mu _{ij}$, the dispersion parameters *φ_ij_* are estimated through an empirical Bayes (EB) procedure as proposed in (11). The procedure borrows information from all CpG sites, and provides more accurate estimates of the dispersion. The EB procedure does not require replicated data as long as *μ_ij_* is available. This makes sense because with the mean methylation known, even one observed data point can provide some information for the variance. Taking advantage of the spatial correlation in methylation levels, the means can be estimated through smoothing. So intuitively, when there is no replicate, data from nearby CpG sites can serve as ‘pseudo-replicates’. We will show below that the procedure works well in both simulation and real data analysis. Although it is still preferable to have biological replicates, DSS-single works better than methods that completely ignore the variance, e.g. methods that use the differences in means or Fisher's exact test to call DMRs.

### DMR calling algorithm

We use the following algorithm for DMR detection from WGBS data. The inputs for the algorithm are *X_ij_*, *N_ij_* and *l_j_*. We first perform local smoothing on estimated methylation proportions to obtain estimates for *μ_ij_*. Next, we estimate the dispersions through the EB procedure described above. We then identify DML (differentially methylated loci) by performing a hypothesis test: $H_0 :\mu _{i1} = \mu _{i2}$ for the equality of mean methylation levels in two groups at each CpG site. To do this, we adopt the Wald test procedure proposed in (11), and modify the variance calculation to account for smoothing effects (details provided in Supplementary Materials). The Wald test is performed at each CpG site, and *P*-values are obtained from the test statistics. Finally, a user-defined *P*-value threshold and additional criteria such as minimum region length are applied to define DMRs.

### WGBS data sets

We analyze several WGBS data sets generated by Roadmap Epigenomics projects (22), including H1 (human embryonic stem cells) and IMR90 (human fibroblasts) cell lines, as well as human liver and hippocampus. The H1 and IMR90 data were obtained from the Gene Expression Omnibus (GEO) with accession number GSE16256, and the hippocampus and liver data are also from GEO with accession number GSE64577. There are two replicates available for each sample, but we limit our analyses to single-replicate comparisons. We perform analyses between H1 versus IMR90, and liver versus hippocampus to evaluate the DMR calling results. The H1 data are also used as template to simulate realistic WGBS data.

For H1-IMR90 comparison, the benchmarks are created as follows. After obtaining the DNase-seq data for H1 and IMR90 from ENCODE data (23), we apply MAnorm (24) to compare them and generate differential DNase I hypersensitive sites (DHSs). CpG island (CGI) data were downloaded from UCSC genome browser (25), with CGI shores defined as ± 1000 base pairs of each side of a CGI. Gene expression for H1 and IMR90 was obtained from (6), and the RPKM values are downloaded from the Human DNA Methylome website at the Salk Institute. We define differentially expressed genes (DEGs) as regions with absolute log2 fold changes of RPKMs greater than 1. The promoter regions for DEGs are defined as the regions ± 5000 base pairs of the transcriptional start sites. The genome segmentation by ChromHMM for H1 cells are obtained from ENCODE.

For liver-hippocampus comparisons, we also utilize available gene expression data and define DEGs using the same approach. However, since the DNase-seq data for these samples are unavailable, differential DHSs cannot be defined. Instead, we use the list of DNase I Hypersensitivity Clusters (DHCs) obtained from ENCODE, which is based on the union of DNase-seq peaks from 125 cell types. We use this list as a benchmark to assess the DMR calling accuracies under the assumption that this list contains active genomic regions for all biological conditions, and thus the DMRs are more likely to overlap with these regions.

### Simulation settings

All simulations are based on WGBS data from the H1 cell line. In comparisons of smoothing procedures and dispersion estimates, we select 20,000 contiguous CpG sites on chromosome 1, smooth the counts using BSmooth with different spans, and treat the smoothed values as the true *μ* in a hypothetical genome region. In each simulation, we generate counts based on the beta-binomial model described above, with *φ* independently generated from the log-normal(−2.5,1) distribution and an average read depth of 10x.

For comparisons of DML and DMR calling in simulations, data are generated for 100,000 CpG sites. We first obtain the true *μ* parameters for the first treatment group by smoothing the data from H1 ESC using BSmooth with smoothing span of 500 bps. We then generate the true *μ* parameters for the second treatment group with 100 DMRs ‘spiked in’ as follows. Using the original H1 data, we randomly generate 100 DMRs with lengths uniformly distributed between 5 and 50 CpGs. To generate these DMRs, we first randomly select 100 regions as ‘target regions’, and then select another set of 100 random regions as ‘source regions’. We obtain the counts (X and N) from the source regions and replace values in the target regions with the counts from the source regions. We then smooth the data as above using BSmooth, and take the results as true *μ* parameters for the second group. We use this approach to guarantee that the true *μ* in the second group is smooth even after spiking in DMRs. Under this simulation setting, about 5% of the CpG sites lie in the DMRs. As above, we then generate counts from the true *μ*'s in each group by independently simulating the dispersion parameters *φ* from a log-normal(−2.5,1) distribution, and then generating the counts based on the beta-binomial model.

## RESULTS

### Smoothing to incorporate spatial information

Smoothing is an integral part of DMR calling because it incorporates information from spatial correlation. We perform simulations to compare smoothing procedures, applying these methods to estimate the mean methylation levels under different scenarios (details in the Materials and Methods section). Table 1 summarizes the correlations of the estimated and true means for each approach.

**Table 1.** Correlation between estimated and true mean methylation levels using different methods and smoothing spans

	True smoothing span 500 bps			True smoothing span 2000 bps		
Smoothing span (bp) used in estimation	500	1000	2000	500	1000	2000
No smoothing	0.966	0.966	0.965	0.960	0.959	0.961
Moving average	0.992	0.973	0.922	0.996	0.995	0.979
BSmooth	0.996	0.989	0.960	0.997	0.998	0.997

Overall, the simulation results show that smoothing increases the accuracy of the mean estimates. When the true smoothing span is 500 bps, correctly specifying the span gives very accurate estimates (correlations > 0.99) when smoothing is performed via either BSmooth or a simple moving average. However when the span is over-specified, correlations drop significantly. For example, using a 2000-bp smoothing span results in correlations of 0.92 and 0.96 correlations for moving average and BSmooth, respectively. On the other hand, when the true smoothing span is 2000 bps, under-specifying the span does not hurt the results as much: results obtained using a 500-bp span have correlations greater than 0.99. It also shows that BSmooth performs slightly better than moving average, especially when the smoothing span is over-specified. However BSmooth is much more computationally intensive, so the slight gain in precision does not justify the computational burden. For these reasons, we elect to take a simple moving average of the collapsed counts for smoothing, and use a conservative smoothing span (500 bps) by default.

### Dispersion estimation to model biological variance

**Figure 1.**
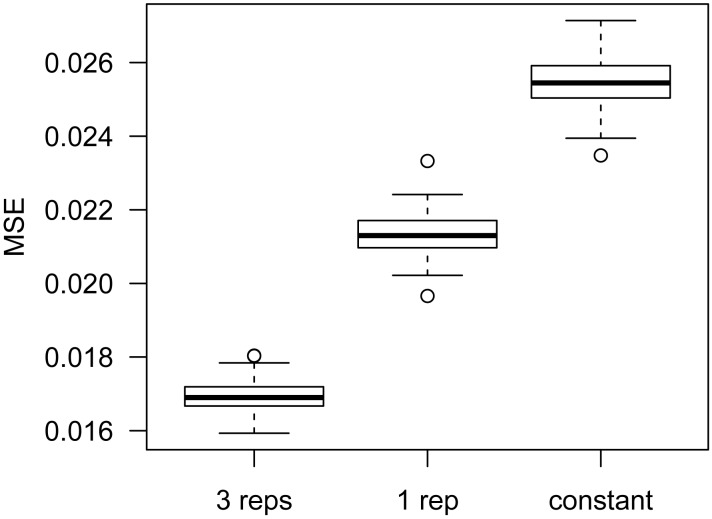
Comparison of dispersion estimates from different methods. For each simulation, MSE of dispersion estimates is obtained. The figure shows the boxplots of MSEs from 100 simulations. Methods compared are: our previously published empirical Bayes estimation procedure (11), using data from three replicates; DSS-single, using data from one replicate; and genome-wide constant dispersion of 0.08.

Accurate estimation of the within group variance is crucial in statistical tests for DMR detection. Existing methods characterize the variance via the dispersion parameter in the beta-binomial model (11,14). However, these methods were developed for data with biological replicates. DSS-single can estimate dispersion for data without replicates, using information from nearby CpG sites. To evaluate the accuracy of dispersion estimates, we simulate data for scenarios with either three or one replicate in each condition (details in Materials and Methods). Dispersions at all CpG sites are estimated using an empirical Bayes (EB) method developed in (11) for data with three replicates, or DSS-single for data with a single replicate. The mean squared errors (MSEs) of the estimates (compared to the true dispersions) are computed as measurements of accuracy. Figure 1 compares the dispersion estimates obtained using three versus one replicate, and a constant dispersion of 0.08, which is the median of all true simulated dispersions. As expected, using three replicates provides the most accurate dispersion estimation. However when there is only one replicate, using DSS-single to estimate dispersion produces much more accurate results than using a genome-wide constant dispersion.

We also explore the effects of sequencing depth on the estimation of dispersion using simulations. Table 2 summarizes the Pearson correlations between estimated and true dispersions under different depths, for scenarios with both three and one replicate per condition. Similar to Figure 1, it shows that dispersion estimates are more accurate with three replicates, but using one replicate yields reasonable results, especially with deeper sequencing depth.

**Table 2.** Pearson correlation between estimated and true dispersions under different average read depth, for three or one replicate in each condition

	Coverage = 10x	Coverage = 20x	Coverage = 30x
1 rep	0.33	0.42	0.46
3 reps	0.55	0.62	0.67

We further evaluate the dispersion estimation from the single-replicate scenario in real data, using the cancer-normal comparison data distributed with the bsseqData Bioconductor package (7). Since the true dispersions are unavailable, we estimated the dispersions using all three replicates from the cancer sample and then use them as the baseline for comparison. We then pick data from each individual replicate and applied DSS-single to estimate dispersions. The Pearson correlations between the three-replicate and the single-replicate estimated dispersions are 0.42, 0.51 and 0.46, respectively. We also calculated the MSEs of dispersion estimates compared to baseline, and compared that with genome-wide constant values (using the median of three-replicate dispersions). The MSE is 0.026 for DSS-single, compared to 0.034 using genome-wide constant values.

All simulation-based and real data results demonstrate that estimation of dispersion in data without biological replicates is possible using DSS-single, and the estimation is reasonably accurate. More accurate dispersion estimation will improve the accuracy and power of downstream DMR detection.

### Comparison of DMR calling from simulation

**Figure 2.**
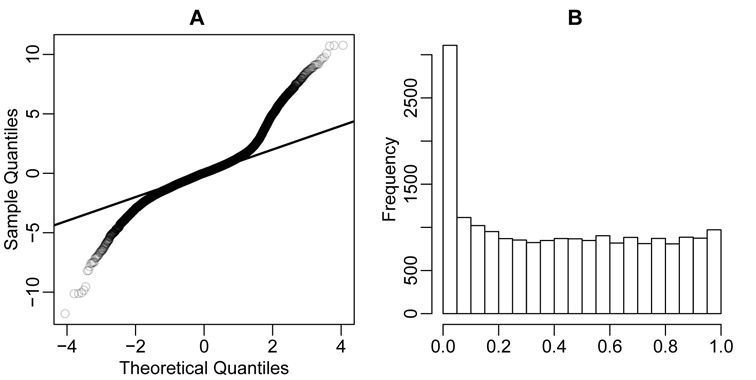
Distributions of test statistics and *P*-values from DSS-single, based on simulation. (**A**) Normal QQ-plot of Wald test statistics. (**B**) Histogram of *P*-values.

We next conduct simulations to compare the results of DMR calling from several methods (see Materials and Methods for simulation details). We first examine the distributions of the test statistics and *P*-values of all CpG sites estimated via DSS-single. Figure 2 shows that the test statistics follow a Gaussian distribution very well under the null hypothesis, and the distribution of *P*-values behaves well with a near-uniform distribution for most values and a spike close to 0 representing the 5% of CpG sites lying within DMRs.

**Figure 3.**
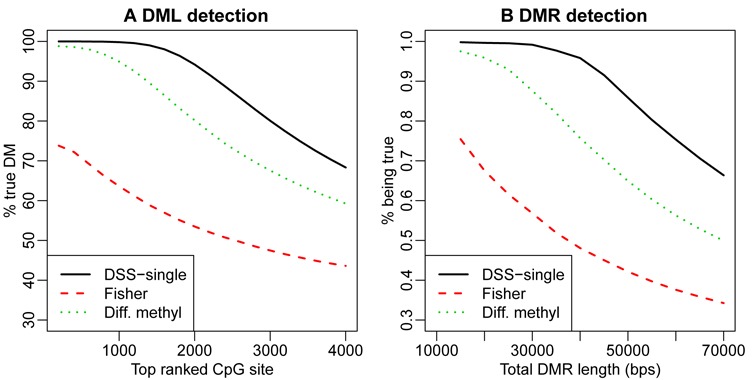
Comparison of DML/DMR calling results from simulation analyses. (**A**) DML calling. X-axis is the number of top-ranked CpG sites considered, where rank is determined according to the reported test statistics or *P*-values. Y-axis is the percentage of CpG sites in the set of top-ranked CpG sites that are truly differentially methylated. (**B**) DMR calling. X-axis is the total length (in bps) of top ranked DMRs called. Y-axis is the percentage (in of bps) of top-ranked DMRs that are truly differentially methylated.

We then compare DMR calling results from several different methods. For most methods, DMRs are detected based on statistical tests performed at each CpG site. The accuracy in calling DML is crucial in the success of DMR calling, so we first evaluate the DML calling results. We compare DSS-single to Fisher's exact test (which is the most straightforward method for data without replicates, and implemented in both methylKit and BSmooth), and the simplistic approach of using the difference of estimated methylation levels (from BSmooth) of two groups. Figure 3(A) compares the true discovery rate (TDR, defined as the percentage of top-ranked CpG sites that are truly DML) of sets of top ranked CpG sites from different methods, showing that DSS-single performs the best in terms of true discovery rate at the CpG site level. We next compare the DMR calling results at the region level. We define the region-level TDR as the percentage (in terms of base pairs) of the called DMRs that are truly differentially methylated, versus the total length of the top ranked DMRs. Figure 3(B) shows the region level TDR curves comparing different methods. Again, DSS-single provides the best results.

### Real data analysis

We next apply our method to public data sets for comparisons between H1 versus IMR90 and liver versus hippocampus. We identify DMRs in both data sets using the four methods currently available for the single-replicate case: DSS-single, methylKit and MOABS (including two DMR lists termed ‘M1’ and ‘M2’ by MOABS), and then compare the DMRs using different criteria.

Extensive public data are available for H1 and IMR90 cell lines that can be used to create benchmarks for method comparison. We evaluate the DMRs from different methods based on their overlap with the following genomic features: (1) Differential DNase I hypersensitive sites (DHSs) from DNase-seq data. DHSs are known to mark active genomic regions such as protein binding sites (26). We have previously observed a strong correlation between DHS and DNA methylation levels, where the peaks from DNase-seq experiment are usually hypomethylated (27). Thus, true DMRs are more likely to overlap with differential DHSs. (2) Promoter regions of differentially expressed genes (DEGs). It is well established that DNA methylation correlates with gene expression (4,28), suggesting that the promoters of DEGs are more likely to be DMRs. (3) CpG island (CGI) shores, since it has been reported that many DMRs are found at CGI shores (29). We create lists of these regions based on public data sets (details in Materials and Methods).

**Figure 4.**
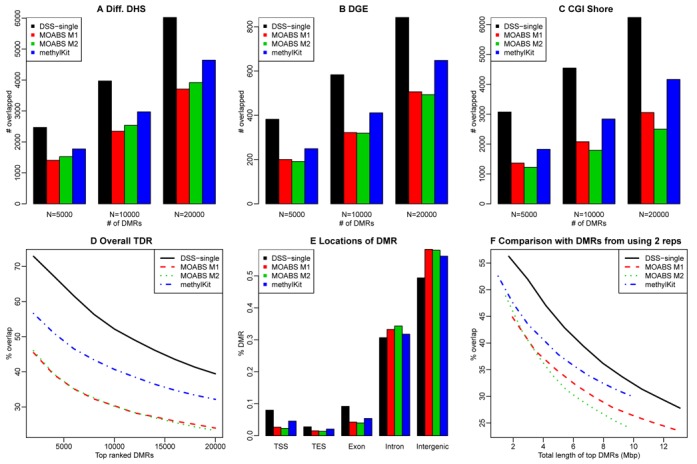
Comparison of DMR calling results for H1 versus IMR90. A-C shows comparisons of sensitivities of different DMRs, where Y-axis shows the number of different genomic features overlapping the DMRs: (**A**) differential DHSs; (**B**) promoter regions of differentially expressed genes; (**C**) CpG island shores. (**D**) Accuracies of top ranked DMRs from different methods. (**E**) Locational distribution of DMRs from H1-IMR90 comparison. Y-axis represents the percentage of DMRs overlapping different genomic features. TSS: transcriptional start site. TES: transcriptional end site. (**F**) Compare with DMRs called from using two replicates. X-axis is the total length of different number of top ranked DMRs. Y-axis is the percentage of overlaps (in terms of base pairs).

We first assess the sensitivity of the DMR calling results. For each of the above genomic features, we count the number covered by the top 5000, 10,000 and 20,000 DMRs, and use these number as measurement for sensitivity. To ensure a fair comparison, we make all DMRs the same length at 300 bps by extending or truncating from the center of the reported DMRs. Figure 4 (A)–(C) show these numbers for DMRs called from different methods. Compared with other methods, the DMRs from DSS-single overlap more differential DHSs, promoters of DEGs and CGI shores. For example, ∼2500 differential DHSs overlap the top 5000 DMRs from DSS-single, compared to ∼1500 for MOABS and ∼1700 for methylKit. Next, we assess the accuracy of DMRs called by different methods. We take the union of all three genomic features, and obtain a list of 134 945 regions occupying a total of 163 Mbp of genome. We use these regions as a gold standard for DMRs, and compute the percentage of top ranked DMRs overlapping these regions. Figure 4(D) shows these percentages, demonstrating that the DMRs from DSS-single are more likely to lie in these regions than other methods. For the top 1000 DMRs from DSS-single about 72% fall in these regions, compared to around 56%, 45% and 46% for methylKit, MOABS M1 and MOABS M2, and 5.4% for randomly selected 300-bp regions.

We also explore the locational distribution of DMRs called by different methods (Figure 4E). Compared with other methods, DMRs from DSS-single are enriched for transcriptional start site (TSS), transcriptional end site (TES), and exonic regions, and relatively depleted in intronic and intergenic regions. Further, we compare the overlap of DMRs with different genome segmentation defined by ChromHMM (30). ChromHMM segments the genome into 15 types of regions. Supplementary Figure S1 shows the number of different regions overlapped with top ranked DMRs. In general, DMRs identified by DSS-single have greater overlap with promoters, enhancers and transcriptional regions, and decreased overlap with heterochromatin and repetitive regions. These results suggest that the DMRs identified via DSS-single tend to lie in more important genomic locations.

We further evaluate the single-replicate DMR calling results by comparing them to the DMRs called using two replicates, since there are two biological replicates for both H1 and IMR90. We call DMRs from two replicates using BSmooth, and use that as our gold standard. Note that it is important to use independent software to generate the gold standard in order to avoid method-specific bias (e.g. DSS-single will be more similar to DSS with two replicates, and MOABS with a single replicate will be more similar to MOABS with two replicates, etc.). Using BSmooth provides an objective gold standard and ensures the fairness in such comparison. We compare the degree of overlap between the gold standard and the single-replicate DMRs called using each method. Figure 4(F) shows the percentage of top ranked DMRs overlapping gold standard DMRs (in terms of base pairs) versus the total length of the top ranked DMRs. DSS-single has the greatest overlapping percentages, suggesting that DSS-single produces results most similar to results obtained using two replicates.

For the liver-hippocampus comparison, we perform similar analyses and evaluations. Since the DNase-seq data are unavailable for these samples, we use the DNase I Hypersensitivity Clusters (DHCs) provided by ENCODE as a proxy for differential DHSs. The DHCs can be viewed as active genomic regions for all biological conditions, and are thus more likely to overlap with DMRs. Although using DHCs is not an ideal proxy for differential DHSs and may overestimate accuracy, it still provides an objective benchmark for evaluating different methods. The results for these analyses are shown in Supplementary Figure S2. As shown in Supplementary Figure S2 (A)–(C), the DMRs from DSS-single have greater overlap with DHC, DEGs and CGI shores compared with other methods. Supplementary Figure S2(D) shows that the accuracy for top ranked DMRs is similar for DSS-single and methylKit and is somewhat lower for MOABS. Supplementary Figure S2(E) shows the locational distribution of DMRs called via different methods, and DMRs from DSS-single again have greater enrichment in TSS, TES and exons. Finally Supplementary Figure S2(F) shows the comparison with the DMRs called from BSmooth using two replicates, and DSS-single again shows the best performance. Overall, these results are consistent with those from H1-IMR90 comparison, and demonstrate the superior performance of DSS-single compared to other methods. Taken together with the simulation results above, DSS-single identifies DMRs with greater sensitivity and accuracy, resulting in a set of findings that is more plausible and interpretable biologically.

### Implementation and computational performance

DSS-single is implemented in the R package DSS, which is freely available on Bioconductor (31). DSS provides excellent computational performance. For 100,000 CpG sites and one replicate in each group, it takes ∼29 s to call DMRs on a Macbook Pro laptop with 2.7 GHz i7 CPU and 16G RAM. The computational time of DSS is linear in the number of CpG sites, so for a typical WGBS data set with 25 million CpG sites, it will take ∼2 h to finish on a computer with similar settings.

## DISCUSSION

Whole genome BS-seq is a new technology for measuring genome-wide DNA methylation. An important goal in WGBS data analysis is the detection of DMRs. Due to the high cost, many WGBS experiments are performed without biological replicates, which makes DMR calling via existing methods difficult or impossible. In this work, we use a hierarchical model to characterize the unique features of data from WGBS experiments, and develop an algorithm for DMR detection for data without replicates. The method considers three important characteristics of the data: spatial correlation between methylation levels from nearby CpG sites, read depth and biological variation. We use a smoothing procedure to capture the spatial correlation of methylation levels, followed by an empirical Bayes shrinkage estimating procedure to estimate the biological variance. Finally, we develop a Wald statistic to provide a formal test for differential methylation, based on our Bayesian hierarchical model that accounts for precision differences due to read depth.

A key feature of DSS-single is to estimate biological variation when replicated data are not available. The method takes advantage of the spatial correlation of methylation levels: since the methylation levels from nearby CpG sites are similar, we can use nearby CpG sites as ‘pseudo-replicates’ to estimate dispersion. By accurately estimating dispersion, DSS-single is able to call DMRs with greater accuracy than simpler approaches based on Fisher's exact test or between-sample methylation differences. To assess the performance of our method, we conducted extensive simulations and analyses of existing WGBS data. The simulation results show that DSS-single provides better dispersion estimates, and hence more accurate DMR calling results. Results from our real data analyses also suggest that the DMR detected from DSS-single are more sensitive and accurate, leading to results that are more consistent with biological expectations.

In comparison to our previously developed DSS method (11), DSS-single has several important distinctions and contributions. First, we adopt local smoothing, and carefully investigate the effects of smoothing method and span. The smoothing procedure is very important and makes the single-replicate dispersion estimation possible. Second, we propose the idea of using nearby CpG sites as ‘pseudo-replicates’ for estimating dispersions, and show by simulation and real data that this approach provides satisfactory results. Moreover, we carefully derive the variance used in Wald statistics for testing DML, with the consideration of smoothing procedure. Finally, we implement the methods into the DSS Bioconductor package, making them easily accessible to the epigenomics research community.

It is important to note that correctly specifying the smoothing span is very important in the estimation of mean methylation levels. Based on our simulation results, over-specifying the span is more harmful than under-specifying; thus, we recommend using a relatively smaller span in smoothing. Exploration of the data shows that the methylation levels contain high- and low-frequency signals, so an adaptive smoothing method (with variable spans) might provide the best solution. However currently available adaptive smoothing methods such as penalized splines (32) are computationally too intensive to be applied to the typical WGBS analysis. Moreover, in data with weaker spatial correlations (e.g. hydroxymethylation data), the smoothness assumption is likely to be too strong. We hope to develop novel methods to account for spatial correlations in different scenarios in our future work.

Although DSS-single was designed and tested for application to WGBS data, it can be used to analyze RRBS data in some genomic regions, depending on CpG density. The estimation of dispersion requires borrowing information from nearby CpG sites, so the procedure can be implemented as long as local smoothing can be performed. When analyzing RRBS data with the DSS package, DSS-single will analyze regions with densely clustered CpG sites, and skip the regions with sparse CpG sites.

Another important consideration in clinical practice is potential sample heterogeneity. Solid tissues such as tumor, whole blood or brain are often a mixture of different cell types. Such mixture can mask methylation signals and weaken the power and accuracy of DMR calling. Even though a number of methods have been developed for estimating the mixing proportion and adjusting for sample heterogeneity in other settings (33–36), rigorous methods for DMR calling for BS-seq data that also consider sample heterogeneity are not yet available. We hope to focus on this important problem in our future work.

In summary, we have developed a novel method for DMR detection in whole genome BS-seq data that can be used when biological replicates are unavailable, and our results from simulations and real data analysis demonstrate the improved performance of the method compared to existing approaches. Our method, DSS-single, is implemented in the latest version of DSS, a computationally efficient R package which is freely available through Bioconductor.

## Supplementary Material

SUPPLEMENTARY DATA
