# Supplementary material for: Detection of differentially methylated regions from whole-genome bisulfite sequencing data without replicates
Source: Nucleic Acids Res. 2015 Jul 15;43(21):e141. doi: 10.1093/nar/gkv715 (PMC4666378; doi:10.1093/nar/gkv715)
Supplement: SUPPLEMENTARY DATA [file supp_43_21_e141__index.html]

Detection of differentially methylated regions from whole-genome bisulfite sequencing data without replicates — Detection of differentially methylated regions from whole-genome bisulfite sequencing data without replicates — SUPPLEMENTARY DATA 

# Detection of differentially methylated regions from whole-genome bisulfite sequencing data without replicates

## SUPPLEMENTARY DATA

- SUPPLEMENTARY DATA
- SUPPLEMENTARY DATA
- SUPPLEMENTARY DATA
