# Supplementary material for: Detection of differentially methylated regions from whole-genome bisulfite sequencing data without replicates
Source: Nucleic Acids Res. 2015 Jul 15;43(21):e141. doi: 10.1093/nar/gkv715 (PMC4666378; doi:10.1093/nar/gkv715)
Supplement: SUPPLEMENTARY DATA [file supp_gkv715_nar-01089-met-k-2015-File006.docx]

**1. Wald test procedure**

Following the notation in the paper, the data model for WGBS-seq data is:

$$X_{ij}|N_{ij}, p_{ij}\sim Binomial (N_{ij},p_{ij})$$

$$p_{ij}|\mu_{ij},\phi_{ij}\sim Beta\left( \mu_{ij},\phi_{ij} \right)$$

$$\phi_{ij}\sim log-normal ( m_{j0}, r_{j0}^{2})$$

$$\mu_{ij}=f_{j}(l_{i})$$

We will perform a hypothesis test $H_{0}:\mu_{i1}=\mu_{i2}$ for each *i*. We first estimate $\mu_{ij}$ with a moving average procedure: $\hat{\mu}_{ij}=\sum_{l\in S_{i}} X_{lj}/ \sum_{l\in S_{i}} N_{lj}$. The variance of the estimate can be derived as follows:

$$var\left( \hat{\mu}_{ij} \right)=\frac{var(\sum_{l\in S_{i}} X_{lj})}{\left( \sum_{l\in S_{i}} N_{lj} \right)^{2}}\equiv\hat{var}_{i1}$$

It is known from the beta-binomial distribution that:

$$var\left( X_{lj} \right)=N_{lj}\mu_{lj}\left( 1-\mu_{lj} \right)\left[ 1+\left( N_{lj}-1 \right)\emptyset_{lj} \right]$$

However, evaluation of $var\left( \sum_{l\in S_{i}} X_{lj} \right)$ is tricky because we need to account for the correlations among $X_{lj}$ from nearby *l*’s, which is not very easy to estimate.

To accomplish this, we assume an AR(1) process of the methylation levels, and assume the lag-1 auto-correlation is $\rho$, that is, $cor\left( p_{ij}, p_{lj} \right)=\rho^{\delta}$, here $\delta=|l-i|$. From the beta distribution, we have: $var\left( p_{ij} \right)=\mu_{i}\left( 1-\mu_{i} \right)\phi_{i} , var\left( p_{lj} \right)=\mu_{l}\left( 1-\mu_{l} \right)\phi_{l}$. Thus the covariance of $p_{ij}$ and $p_{lj}$ is:

$$cov\left( p_{ij}, p_{lj} \right)=\rho^{\delta}\sqrt{\mu_{i}\left( 1-\mu_{i} \right)\phi_{i}\mu_{l}\left( 1-\mu_{l} \right)\phi_{l}}$$

Based on this, we can compute the covariance of $X_{ij}$ and $X_{lj}$ as follows:

$$cov\left( X_{ij}, X_{lj} \right)=E\left[ X_{ij} X_{lj} \right]- E\left[ X_{ij} \right]E[X_{lj}]$$

$E\left[ X_{ij} X_{lj} \right]$ can be calculated using the iterative expectation procedure:

$$E\left[ X_{ij} X_{lj} \right]= E\left\{ E\left[ X_{ij} X_{lj} | p_{ij},p_{lj} \right] \right\}=N_{ij}N_{ij}E[p_{ij}p_{lj}]$$

Because we have:

$$E\left[ p_{ij}p_{lj} \right]= cov\left( p_{ij}{,p}_{lj} \right)+E\left[ p_{ij} \right]E\left[ p_{lj} \right]$$

$$= \rho^{\delta}\sqrt{\mu_{i}\left( 1-\mu_{i} \right)\phi_{i}\mu_{l}\left( 1-\mu_{l} \right)\phi_{l}}+\mu_{i}\mu_{l}$$

then

$E\left[ X_{ij} X_{lj} \right]=N_{ij}N_{lj}\left[ \rho^{\delta}\sqrt{\mu_{i}\left( 1-\mu_{i} \right)\phi_{i}\mu_{l}\left( 1-\mu_{l} \right)\phi_{l}}+\mu_{i}\mu_{l} \right]$,

and

$$cov\left( X_{ij}, X_{lj} \right)=N_{ij}N_{lj}\left[ \rho^{\delta}\sqrt{\mu_{i}\left( 1-\mu_{i} \right)\phi_{i}\mu_{l}\left( 1-\mu_{l} \right)\phi_{l}}+\mu_{i}\mu_{l} \right]- {N_{ij}\mu}_{i}N_{lj}\mu_{l}$$

$$= N_{ij}N_{lj} \rho^{\delta}\sqrt{\mu_{i}\left( 1-\mu_{i} \right)\phi_{i}\mu_{l}\left( 1-\mu_{l} \right)\phi_{l}}$$

With the variance/covariance matrix of ${[X}_{lj}, l\in S_{i}]$, $var\left( \sum_{l\in S_{i}} X_{lj} \right)$ can be directly computed using the standard procedure.

The estimation of $\rho$ is based on all data from the whole genome using the standard procedure. Estimated values of $\rho$ were all around 0.8 in several WGBS datasets tested.

Once the variances are estimated, the Wald test statistics can then be computed as:

$$t_{i}=\frac{\hat{\mu}_{i1}-\hat{\mu}_{i2}}{\sqrt{\hat{var}_{i1}+\hat{var}_{i2}}}$$

**2. Overlaps of DMRs with ChromHMM segmentation of the genome**

Figure S1: Number of different ChromHMM regions overlapping with DMRs called from several methods.

**3. Analysis results for liver vs. hippocampus comparison**

Figure S2: Comparison of DMR calling results for liver vs. hippocampus. A-C shows comparisons of sensitivities of different DMRs, where Y-axis shows the number of different genomic features overlapping the DMRs: (A) DHC (DNase I Hypersensitivity Clusters); (B) promoter regions of differentially expressed genes; (C) CpG island shores. (D) Accuracies of top ranked DMRs from different methods. (E) Locational distribution of DMRs. Y-axis represents the percentage of DMRs overlapping different genomic features. TSS: transcriptional start site. TES: transcriptional end site. (F) Comparison with DMRs called using two replicates. X-axis is the total length of different number of top ranked DMRs. Y-axis is the percent overlap (in terms of base pairs).
