# Supplementary figures and images for: Detection of differentially methylated regions from whole-genome bisulfite sequencing data without replicates
Source: Nucleic Acids Res. 2015 Jul 15;43(21):e141. doi: 10.1093/nar/gkv715 (PMC4666378; doi:10.1093/nar/gkv715)

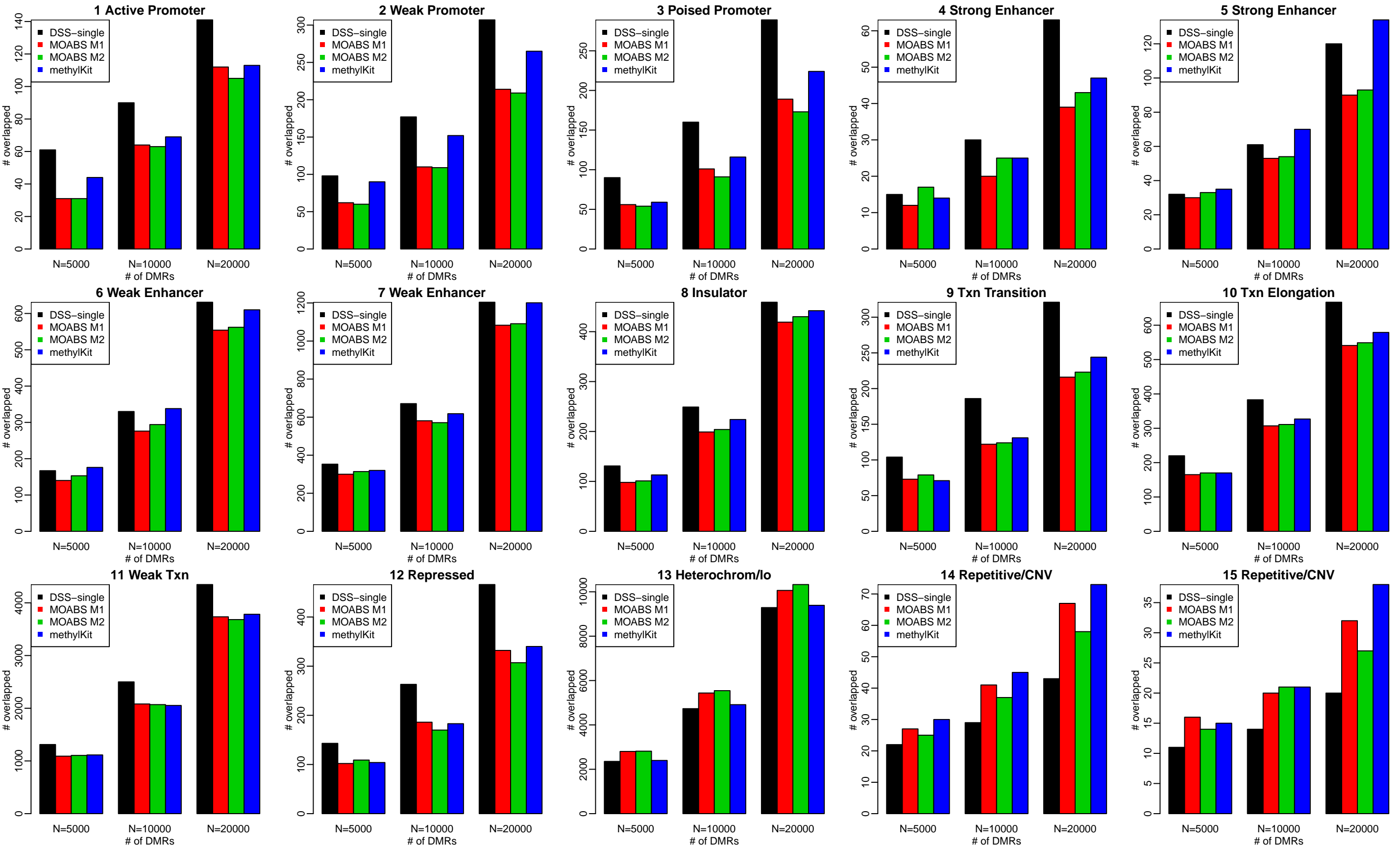

Supplement: SUPPLEMENTARY DATA [file supp_gkv715_nar-01089-met-k-2015-File007.pdf]

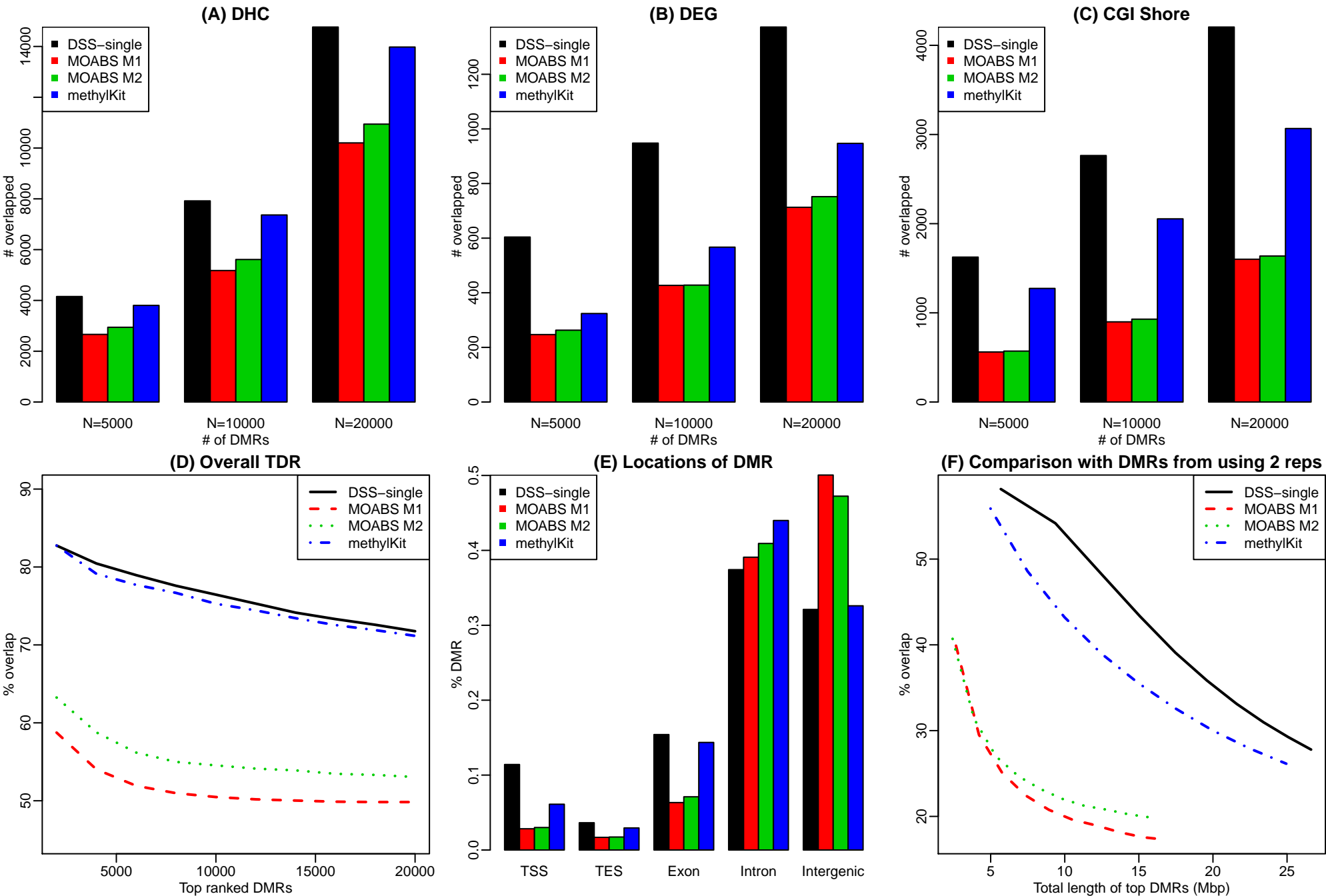

Supplement: SUPPLEMENTARY DATA [file supp_gkv715_nar-01089-met-k-2015-File008.pdf]
